# Perspective: Why Whole Grains Should Be Incorporated into Nutrient-Profile Models to Better Capture Nutrient Density

**DOI:** 10.1093/advances/nmaa172

**Published:** 2021-01-28

**Authors:** Adam Drewnowski, Nicola McKeown, Katrina Kissock, Eleanor Beck, Heddie Mejborn, Florent Vieux, Jessica Smith, Gabriel Masset, Chris J Seal

**Affiliations:** Center for Public Health Nutrition, University of Washington, Seattle, WA, USA; Jean Mayer USDA Human Nutrition Research Center on Aging at Tufts University, Boston, MA, USA; Friedman School of Nutrition Science and Policy, Tufts University, Boston, MA, USA; School of Medicine, Faculty of Science, Medicine, and Health, University of Wollongong, Wollongong, New South Wales, Australia; Illawarra Health and Medical Research Institute, University of Wollongong, Wollongong, New South Wales, Australia; School of Medicine, Faculty of Science, Medicine, and Health, University of Wollongong, Wollongong, New South Wales, Australia; Illawarra Health and Medical Research Institute, University of Wollongong, Wollongong, New South Wales, Australia; National Food Institute, Technical University of Denmark, Kongens Lyngby, Denmark; MS-Nutrition, Marseille, France; General Mills Scientific and Regulatory Affairs, Minneapolis, MN, USA; Cereal Partners Worldwide, Prilly, Switzerland; Public Health Sciences Institute, University of Newcastle, Newcastle upon Tyne, United Kingdom

**Keywords:** dietary guidelines, whole grains, cereal fiber, nutrient profiling, Nutri-Score, Nutrient Rich Food index, Health Star Rating, energy density, nutrient density, food groups

## Abstract

Healthy eating patterns, as described by dietary guidelines, typically favor whole grains, low-fat dairy, vegetables, fruit, legumes, and nuts and seeds. Nutrient-profiling (NP) models capture nutrient density of individual foods and can inform healthier food choices. Although whole grains are prominently featured in most dietary guidelines, they are not included in most NP models. Healthy foods, as identified by most NP models, are those that contain limited amounts of energy, saturated fat, total or added sugar, and sodium. As global dietary guidance turns to foods and food groups as opposed to individual nutrients, future nutrient-density metrics may need to do the same. Potential methods to incorporate whole grains into the overall concept of nutrient density and into selected NP models are outlined in this review. Incorporating whole grains into the Nutri-Score, Health Star Rating, or the Nutrient Rich Food index will require further analyses of dietary nutrient density in relation to health outcomes across diverse population subgroups. We present the rationale for how the inclusion of whole grains in NP models can assist in the implementation of dietary guidance.

## Introduction

Dietary guidelines, intended to promote more healthful and nutrient-dense diets, are the chief instrument of national food and nutrition policies worldwide (1–6). While continuing to stress the need to limit saturated fats, added sugar, and sodium, most dietary guidelines are becoming increasingly food-based (1–6). The current dietary guidelines in the United States (1), Australia (2), New Zealand (3), and countries in the European Union (4–6) have become more focused on the observed links between recommended food groups, dietary patterns, and multiple health outcomes (1–6).

The place of whole grains in healthy eating patterns is already well established (7). The Dietary Guidelines for Americans (DGA), reissued every 5 y (8–10), have featured whole grains since the 2000 edition. The 2005 DGA (9) was the first to recommend that whole grains be consumed daily (9). The 2015–2020 DGA (11, 12) provides advice on how diets composed of whole grains, vegetables, fruit, low-fat dairy, legumes, and nuts and seeds can meet nutrient requirements and lower disease risk. Dietary guidelines in Australia (2), New Zealand (3), and the European Union (4–6) have also stressed the importance of including whole grains along with vegetables and fruit in healthy eating patterns.

The 2020 Dietary Guidelines Advisory Committee (1) identified whole grains, vegetables, and fruit as the 3 plant-based fundamental constituents of a healthy dietary pattern. The advice was to consume at least 3 ounce-equivalents of whole grains per day in a 2000-kcal diet (12). The Eat for Health Australian Dietary Guidelines (2) advised choosing mostly whole-grain and/or high-fiber versions of grain foods. The Eatwell Guide (5) for the United Kingdom advised choosing whole-grain foods where possible. The French guidelines were to consume 50% of all grains as whole grains and to consume them daily (6).

Whole grains are defined by the US FDA as “the intact, ground, cracked, flaked or otherwise processed kernel after the removal of inedible parts such as the hull and husk. All anatomical components, including the endosperm, germ, and bran must be present in the same relative proportions as in the intact kernel” (13). Similar definitions are used by Food Standards Australia New Zealand (14) and by the Whole Grain Initiative (15) and the Health Grain Forum (16).

Definitions of “whole-grain rich” foods can vary (17, 18) and regulations as to the minimum content of whole grains are not always in place. In the United States (12), a food is 100% whole grain if whole grains are the only grains that it contains. One ounce-equivalent of whole grains can contain between 16 and 28 g of whole grains, depending on product type. The US National School Breakfast and Lunch Program (19) requires whole-grain rich foods to contain 51% or more whole-grain ingredient(s) by weight per reference amount customarily consumed (RACC). In Sweden, foods must be >50% whole grain to be labeled whole-grain foods (20). The Healthgrain Forum (16) has proposed >30% based on product dry weight and exceeding the content of refined grains. However, no accepted global definition of whole-grain rich foods exists at this time.

Effective dietary guidance to promote whole-grain consumption may require a more unified approach (18). In particular, whole-grain content of foods could be better captured by nutrient-profiling (NP) methods that serve as the basis for nutrition and health claims. A glossary of terms is provided in **Table 1**. Outlining the rationale and ways to incorporate whole grains into quantitative NP models of nutrient density is the topic of this review.

**TABLE 1:** Glossary of terms

	Definition
Whole grains	The FDA defines whole grains as “the intact, ground, cracked, flaked or otherwise processed kernel after the removal of inedible parts such as the hull and husk. All anatomical components, including the endosperm, germ, and bran must be present in the same relative proportions as in the intact kernel.”
Dietary fiber/cereal fiber	Carbohydrate polymers with ≥3 monomeric units, which are not digested or absorbed in the human small intestine and may be naturally occurring, isolated, or synthetic.
Ready-to-eat cereals	Sometimes referred to as “cold” breakfast cereals and can include both whole-grain and refined-grain varieties.
Dietary patterns	Refers to the quantities, proportions, variety or combinations of different food and beverages in diets, and the frequency with which they are consumed.
Nutrient density	Nutrient content per reference amount of food per 100 g, 100 kcal, or serving.
Nutrient profiling	Quantitative methods to capture nutrient density of individual foods, but also meals and the total diet.
Food groups and categories	A collection of foods that are classified in the same food category (e.g., dairy, meat, grains) or grouped together because they share similar nutritional properties.
Nutrient Rich Food index	A formal scoring system ranking foods based on their nutrient content; can be used together with food prices to aid in the identification of foods that are both nutritious and affordable.
Nutri-Score	Front-of-pack labeling system first adopted in France in 2017 based on 5 colors and letters (from green/A to red/E); allows consumers to identify at a glance the nutritional value of prepackaged foods.
Health Star Rating	Front-of-pack labeling system developed in Australia rating the nutritional profile of packaged food on a scale from ½ a star to 5 stars; provides a standard way to compare similar packaged foods—the more stars, the healthier the choice.
Nutrient-composition databases	Databases that contain data on energy, energy-yielding nutrients, fiber, vitamins, minerals, and phytochemicals.

## Current Status of Knowledge

Whole-grain consumption in most countries remains low. In France (21) and Italy (22), half of all adults typically consumed no whole grains, and median intakes were therefore zero. Median whole-grain intakes among adults in Australia (23) and in the United Kingdom (24, 25) were only 20 g/d. Analyses of national trends in the United States for the period 2001–2010 (26) showed minimal increases in whole-grain intake. Only Scandinavian countries showed higher consumption of whole grains (27–29). In the Danish Diet, Cancer and Health–Next Generations cohort, 54% of the participants consumed the recommended 75 g/10 MJ of whole grains (27), and the median intake was 79 g/10 MJ. Promoting whole-grain consumption is a global challenge (30).

### Evidence supporting health benefits of whole grains

The Global Burden of Disease Study (31) showed that low intake of whole gains, low intake of fruit, and high intake of sodium accounted for >50% diet-related deaths and a substantial proportion of disability-adjusted life-years related to cardiovascular disease. Meta-analyses of prospective cohort studies have consistently shown a protective association between diets high in whole grains and hypertension (32); type 2 diabetes (33); cardiovascular disease risk (34); colon (35), gastric, and esophageal cancers (36); and all-cause mortality (37). In some observational studies, a higher intake of whole grains was linked to reduced risk of obesity or weight gain (38). Whole grains have also been associated with improved lipid profiles (39), glucose metabolism (39), lower blood pressure (39, 40), and better inflammatory status (41).

Whole grains along with cereal dietary fiber may have an important role in gut health and in the modulation of gut microbiota (42). The soluble fiber β-glucan found in whole-grain oats and barley has an established lipid-lowering effect (39, 43). In observational studies, intakes of both whole grains and cereal fiber were linked to improved cardiovascular risk factors (40–42). However, some health effects of whole grains may go beyond cereal fiber (44–46), as suggested by statistical adjustment for cereal fiber content (41, 43, 44).

Compared with refined flours and foods, whole grains contain more iron, magnesium, manganese, phosphorus, potassium, selenium, and zinc, and vitamins B and E (46). Whole grains also contain polyphenolic phytochemicals (47, 48), such as phenols, flavonoids, and carotenoids (lutein, zeaxanthin, and β-cryptoxanthin). These compounds may act as antioxidants in the gut, or have direct cell-signaling effects if metabolized and absorbed. The majority of these nutrients and phytochemicals are located in the aleurone, bran, and germ layers of the intact cereal grain and are removed from the grain during milling to produce refined (white) flours (46).

Higher nutrient density of whole grains may help explain some of the observed health benefits. Meta-analysis of up to 22 randomized clinical trials (49) showed that replacing refined grain or placebo with whole grain led to improvements in plasma lipid profiles that were attributed to whole-grain oats. Replacing refined grains with whole grains also improved glycated hemoglobin (a marker of diabetes risk) and C-reactive protein (a marker of inflammation). Given the complexity of the mechanistic pathways involved, it is unlikely that the observed cardioprotective benefits attributed to whole grains are due solely to a single isolated nutrient. Rather, the health value of whole grains lies in the complex interplay between dietary fiber, minerals, vitamins, compounds, phytosterols, and many other bioactive components. The study of the microbiome and the metabolome may provide further insights into the health benefits of whole grains and cereal fiber.

#### Evidence supporting whole-grain nutrition and health claims

Recognizing the need to promote whole-grain consumption, the FDA has provided guidance to industry regarding whole-grain label statements since 2006 (13, 50). Manufacturers are permitted to make factual statements about whole grains on the food label (e.g., 100% whole grain) provided that the statements are not false or misleading and do not imply a particular level of ingredient (e.g., high or excellent source) (13). Manufacturers may also use health claims relating whole grains to a reduced risk of coronary heart disease and certain cancers. The preferred statement reads: “Diets high in plant foods—i.e., fruits, vegetables, legumes, and whole grain cereals—are associated with a lower occurrence of coronary heart disease and cancers of the lung, colon, esophagus, and stomach” (50). Other regions such as the European Union, Australia, and New Zealand do not allow whole-grain health claims.

Regulatory agencies have also made some efforts to define the term “healthy” that is generally based on the nutrient content of foods (51). Foods that exceed prespecified contents of total fat or saturated fat, total or added sugars, and sodium can be disqualified (51). In Australia, such foods are classified as discretionary and therefore unable to display a health claim, regardless of other nutrients they may contain (52). The citizen petition submitted in 2015 by KIND LLC to the FDA (53) argued that these purely nutrient-based requirements were reductionist and no longer supported by current science. The argument that received cross-sector support (54, 55) was that the food's content of nuts, grains, seeds, and other food groups aligned better with its overall nutritional value than did the specified saturated fat content of <1 g/serving. Amending the definition of healthy foods to include whole grains could be a powerful health policy tool (55).

### Whole grains are included in diet-quality metrics

Measures of overall diet quality are typically based on the degree of adherence to dietary guidelines. The USDA Healthy Eating Index (HEI) 2015 (56) is a 100-point score of diet quality that is reissued with each new edition of the DGA (57–60). The original HEI-1995 (57) was largely nutrient based, with the major scoring components based on total fat, saturated fat, total cholesterol, sodium, and dietary variety. In keeping with the trends in dietary guidelines, measures of diet quality that were formerly nutrient driven have become more food oriented. By 2005 (58), the HEI-2005 included scores that featured total and whole fruit, total vegetables, dark-green/orange vegetables, grains, milk and dairy, meat and beans, and healthy oils. Total scores were reduced by points related to empty calories from solid fats, alcohol, and added sugar (58). The HEI-2010 (59) awarded points for total and whole fruits, total vegetables, greens and beans, whole grains, milk/dairy, total, seafood and plant protein, and the healthy fat ratio. The HEI-2015 (60) maintained the distinction between whole and refined grains. Refined grains are now moved to the debit side of diet quality, along with sodium, and with empty calories from added sugar and saturated fats.

In parallel to quantitative assessments of diet quality, the goal of NP models is to capture the overall nutritional value of individual foods (61, 62, 64–68, 72, 75, 76). The 2010 WHO report (76) specifically stated that “nutrient profile models need to complement and support food-based dietary guidelines.” Despite the long-standing inclusion of whole grains in global dietary guidelines, most NP models remain purely nutrient based. We need a better alignment between food-based dietary guidelines (70, 77–79) and nutrient-based NP methodologies.

### Most NP models do not capture whole grains

NP models’ estimates of overall nutritional value are based on energy and nutrient content (64–68, 76). The characteristics of selected NP models are summarized in **Table 2**. Among well-known NP models are the Health Star Rating (HSR) (68), Choices International (64), Nutri-Score (67), and the NRF index (61, 62, 75).

**TABLE 2:** Characteristics of selected NP models by category^1^

Model type (reference)	Unit	Positive score based on	Negative score based on	Food groups included in score	Whole grains?
Across the board					
NRF9.3 (61, 62)	kcal	Protein, fiber, Ca, Fe, K, Mg, vit A, C, D	SF, added sugar, Na	No	No
SAIN, LIM (63)	kcal, g	Protein, fiber, Ca Fe, vit A, C (D optional)	SF, added sugar, Na	No	No
Category specific					
Choices (64)	g	Fiber	SF, added sugar, Na	No	No
Keyhole (65)	g	Fiber	TF, SF, total + added sugar, Na	Whole-grain foods are a category	Yes
Hybrid NP models					
Ofcom 2004^2^ (66)	g	Protein fiber	Energy, SF, total sugar, Na	Fruit, vegetables, nuts,	No
Nutri-Score (67)	g	Protein, fiber	Energy, SF, total sugar, Na	Fruit, vegetables, nuts, legumes, oils	No
HealthStar (68, 69)	g	Protein, fiber	Energy, SF, total sugar, Na	Fruit, vegetables, nuts, legumes	No
NRF6.3 hybrid (70)	kcal	Protein, fiber, K, Ca, Fe, vit D	SF, added sugar, Na	Fruit, vegetables, nuts, legumes, whole grains, oils, dairy, seafood	Yes
NRFh4:3:3 (71)	kcal	Protein, fiber, K, PUFA+MUFA	SF, added sugar, Na	Fruit, dairy, whole grains	Yes
NRFh3:4:3 (71)	kcal	Fiber, K PUFA+MUFA	SF, added sugar, Na	Fruit, dairy, whole grains, nuts + seeds	Yes
SENS (72, 63)	kcal, g	Protein, fiber	SF, added sugar, Na	Fruit, vegetables	No
Negative score only					
PAHO (73)	kcal	None	TF, SF, *trans* fat, Na, free sugar	No	No
Chile (74)	g	None	Added sugar, SF, Na	No	No
WHO (66)	g	None	Energy, TF, SF, *trans* fat, total + added sugar, LCL, Na	No	No

1 LCL, low-calorie sweeteners; LIM, Limit; NP, nutrient-profiling; NRF, Nutrient Rich Food; PAHO, Pan American Health Organization; SAIN, Healthy (French), SENS, Simplified Nutrient Profiling System; SF, saturated fat; TF, total fat; vit, vitamin.

2 Ofcom 2004, formerly called FSA-Ofcom, is the basis for other NP models including Nutri-Score and Health Star Rating.

While far from uniform, NP models do share some common features (Table 2). The basis of calculation has been 100 g (mostly European Union), 100 kcal, or serving size. NP models may be across-the-board or category specific, with different criteria applied to food categories or food groups. Nutrients to encourage typically include protein, fiber, and a range of vitamins and minerals (62). Nutrients to limit typically include total or saturated fat, total, added or free sugar, and sodium (62). NP models can be based on nutrients to limit only or on some combination of beneficial nutrient to encourage and nutrients to limit. Nutrient standards are based on regulatory values and/or dietary guidance.

The original NRF9.3 nutrient-density score (61, 62) was a purely nutrient-based across-the-board NP model. Protein, fiber, vitamin A, vitamin C, vitamin E, calcium, iron, potassium, and magnesium were the 9 nutrients to encourage. Saturated fat, added sugar, and sodium were the 3 nutrients to limit. All nutrients were expressed as percentages of daily values (%DV), all expressed per 100 kcal of food.

Some models did consider both nutrients and ingredients, consistent with the later KIND petition. The Food Standards Agency–Office of Communications (FSA-Ofcom) model (66), now known as Ofcom 2004, awarded points for a food's content of fruit, vegetables, and nuts. The French derivative, Nutri-Score (67), awards points for fruit, vegetables, legumes, nuts, and healthy oils. The Simplified Nutrient Profiling System (SENS) (72) algorithm lists fruit and vegetables. The Australian HSR (68) also awards points for fruit, vegetables, legumes, and nuts. The HSR can be viewed as category specific since different criteria are applied to milk and dairy products. The Choices model (64) has always been category specific, distinguishing between basic and nonessential foods.

The Keyhole label used in most Nordic countries is also category specific (65). The Keyhole label is based on lower and healthier fat, less sugar, less salt, and more fiber and whole grain, all calculated per 100 g of food. The Keyhole system calls out whole grains, which need to constitute from 30% to 100% of the food dry weight, depending on the food group (65). So do new NRF nutrient-density models that include both nutrients and food groups (71).

In contrast, the contribution of whole grains to the NRF index (61), Choices (64), Nutri-Score (67), and HSR (68) models is reduced to the food's fiber content. Using fiber as the main proxy for whole grains seems inconsistent with the current spirit of global dietary guidance, which favors both fiber and whole grains (44–46). Whole grains could be (but are not) on the Nutri-Score list of desirable food ingredients, along with vegetables and fruit. Neither the Nutri-Score nor the HSR specifically addresses the whole-grain content of foods.

### How does Nutri-Score perform with grains and cereals

In the present proof-of-concept analyses, the Nutri-Score algorithm was applied to grains and cereals in Australian and US nutrient-composition databases. First, the Australian Food, Supplement, and Nutrient Database (80–82) for 499 grains and cereals was linked with the Australian whole-grain database (82). The whole-grain content was estimated based on product packaging, whole-grain content claims, ingredient lists, a recipe-based approach, and manufacturer information and calculated on a dry-weight basis. The US analyses used grains and cereals in the USDA Food and Nutrient Database for Dietary Studies (FNDDS) (83). Baby cereals were excluded in both sets of analyses.

The Nutri-Score negative score (N) is based on energy, total sugar, saturated fat, and sodium, while the positive (P) score is based on fiber, protein, and the fruit, vegetable, and nut content. Lower scores denote higher-quality products. However, the N-P calculation in Nutri-Score applies only when N is less than 11 points. Further, protein content matters only when the fruit, vegetable, legume, and nut (FVN) points equal to or exceed 5. For that to happen, the food needs to contain 80% of fruit, vegetables, legumes, or nuts by weight. Since grains and cereals do not generally contain significant amounts of legumes, nuts, vegetables, or fruit, only fiber went into the P score in most cases.

The Nutri-Score awards 0 points to foods with <0.9 g/100 g fiber and a maximum of 5 points for foods with >4.7 g/100 g fiber by weight. The HSR system ranges from 0 (for <0.9 g fiber/100 g) to 15 (for >20 g fiber/100 g of the food) points. In the HSR the calculation is always N-P where P = fruit/vegetables, protein, and fiber (84). The Nutri-Score points were then translated to a letter grade. Scores ≤ −1 translate to A, scores 0–2 become B, scores 3–10 become C, scores 11–18 become D, and scores ≥19 become E.

**FIGURE 1:**
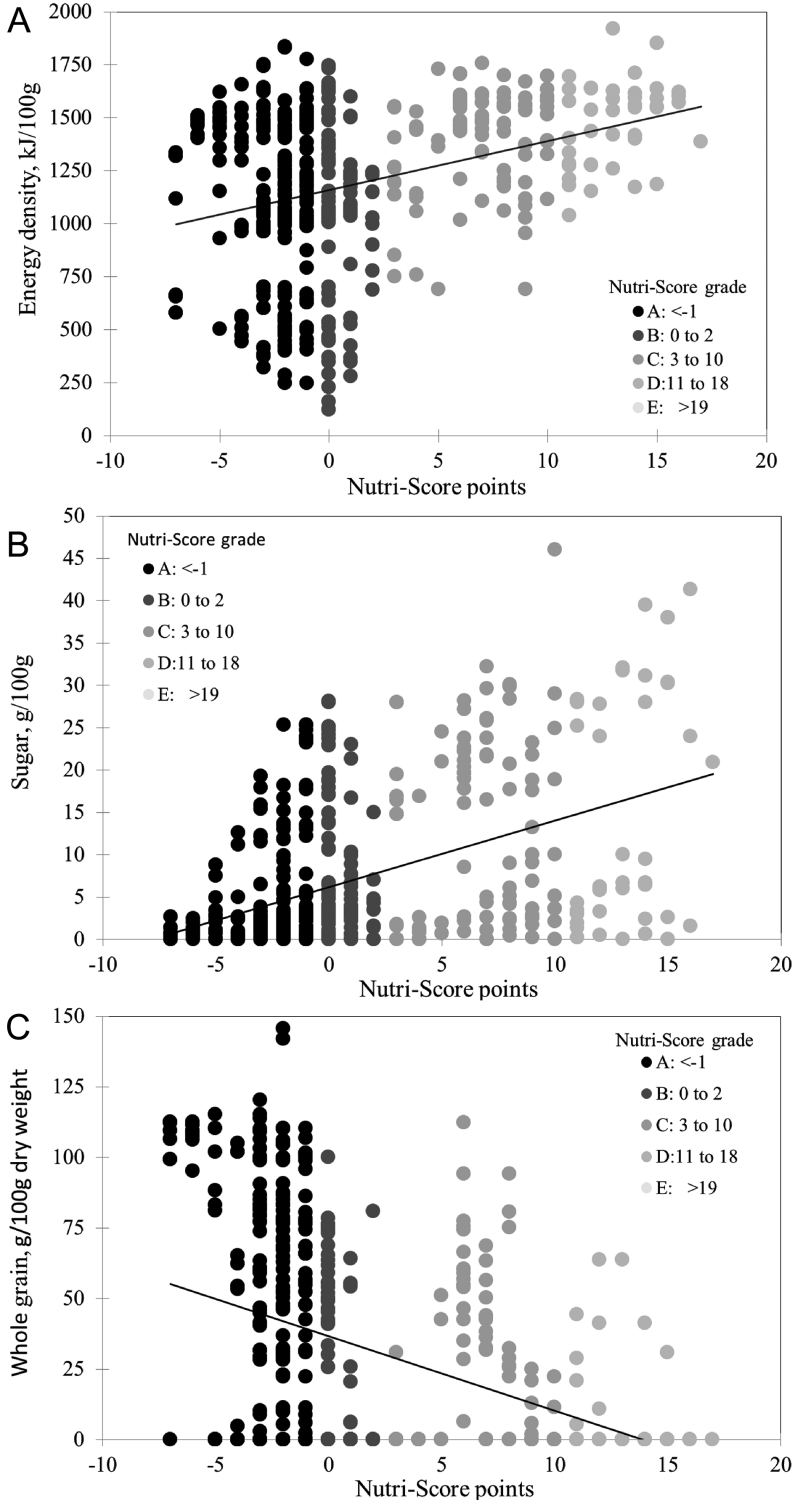
Relations between Nutri-Score points and energy density of grains and cereal products in kilojoules per 100 g (A), total sugar content of grains and cereal products in grams per 100 g (B), and whole-grain content of grains and cereal products in grams per 100 g (C) in the Australia nutrient-composition database (*n* = 499).

**Figure 1**A shows relations between Nutri-Score points and energy density of grains and cereal products in kilojoules per 100 g. Cooked cereals had a low energy density, due to water content, whereas dry cereals had a mean energy density of ∼1500 kJ/100 g. Figure 1B shows the relation between Nutri-Score points and total sugar content of cereal products in grams per 100 g. Nutri-Score points increased with rising sugar content. In general, Nutri-Score values were driven by energy density of foods, given that the overall Nutri-Score score was heavily weighted by energy, sugar, and saturated fat.

Figure 1C shows relations between Nutri-Score points and whole-grain content of cereal products in grams per 100 g. The whole-grain content per dry weight (81) was estimated as described above (82). Although many whole-grain cereals did receive favorable ratings, the reason was lower sugar content as opposed to higher whole-grain content. Within these cereal groups there was no relation between whole-grain content and Nutri-Score.

**FIGURE 2:**
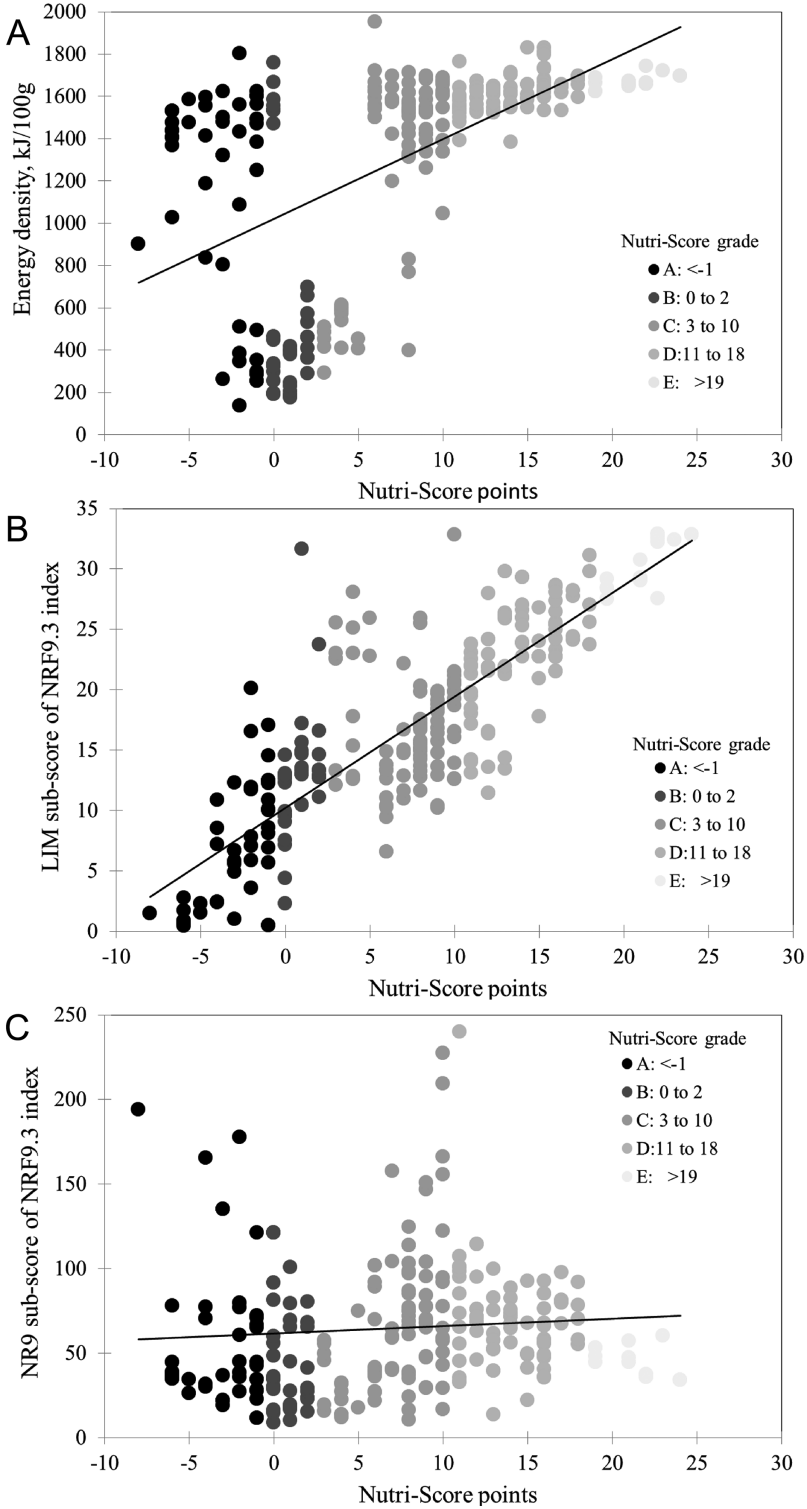
Relations between Nutri-Score points and energy density of grains and cereal products (A), the LIM subscore of the NRF index, based on saturated fat, added sugar, and sodium and calculated per 100 kcal (418 kJ) (B), and the NR9 subscore (C) in the USDA nutrient-composition database (*n* = 265). LIM, Limit subscore; NR9, Nutrient Rich subscore.

**Figure 2**A shows the same relations between Nutri-Score points and energy density of grains and cereal products (kilojoules/100 g) in the USDA FNDDS database. The correlation was 0.54 (*P* < 0.001). Cooked cereals had low energy density, whereas dry ready-to-eat cereals had higher mean energy density.

Figure 2B shows relations between Nutri-Score points and the negative Limit (LIM) subscore of the NRF index. The negative LIM subscore is based on saturated fat, added sugar, and sodium, all calculated per 100 kcal. The correlation was 0.83 (*P* < 0.001). In contrast, as indicated in Figure 2C, the Nutri-Score did not capture the full nutrient density of cereals, especially those fortified with vitamins and minerals. The Nutri-Score was significantly correlated with total sugar (*r* = 0.82) and with fiber (*r* = −27) but not with NRF9.3 nutrient-density score. Several fortified ready-to-eat cereals received high NRF scores. Fortification was not captured by the Nutri-Score.

### How to integrate whole grains in NP models

Both dietary guidelines and NP models are used for educational, regulatory, and policy purposes (83). Whereas dietary guidelines are increasingly focused on food patterns, NP models serve mostly to assess the nutrient density of individual foods. NP models have provided the scientific basis for regulatory and educational initiatives and for product (re)formulation by the food industry (84). Aligning NP scores for individual foods with the broader principles of dietary guidance, as embodied in the DGA, would be a valuable addition to public health–promotion activities.

However, some discrepancies between the food-based approach to dietary guidance and the inherently nutrient-based NP methods need to be resolved. One approach may be to develop new hybrid nutrient-density scores that combine nutrients and selected food groups (70). For example, in standard NRF models, brown rice and white rice had similar NRF6.3 scores. Adding whole-grain points to brown rice raised NRF values. Similarly, whole-wheat bread was ranked higher in the hybrid NRF6.3 model (from 20 to 72), whereas white bread did not (from 12 to 15). Fortified whole-grain, ready-to-eat cereal scored particularly well (from 73 to 102).

New hybrid NP models may align better with food-based measures of a healthy diet. In 1 recent study (71), iterative regressions linked NRF scores based on 16 nutrients and 5 food groups to HEI-2015 values for 23,643 persons aged >2 y in the 2011–2016 NHANES. Nutrient-based NRF scores accounted for up to 66% of the variance, whereas scores based on food groups accounted for 50%. In contrast, the NRF3:4:3 model based on fiber, potassium, PUFAs + MUFAs, whole grains, dairy, fruit, nuts, and seeds explained 72% of the variance. A related NRFh4:3:3 model based on protein, fiber, potassium, PUFAs + MUFAs, whole grain, dairy, and fruit also explained 72% (71). The final hybrid NRF algorithm combined nutrients to encourage (NRx subscore), MyPlate food groups to encourage (MPy subscore), and the 3 nutrients to limit (LIMz subscore).

## Conclusions

Dietary guidelines worldwide are becoming increasingly food based. In the United States, the 2015–2020 DGA favor healthy food patterns built around whole grains, vegetables, fruit, nuts, and a variety of protein sources. The goal of NP models is to promote the implementation of dietary guidance. Yet, many NP models seem to capture energy density rather than nutrient density of foods and most remain purely nutrient based. Those NP models that incorporate beneficial ingredients or food groups still fail to include whole grains, despite considerable evidence linking higher whole-grain consumption with improved health outcomes (85). Modifying NP models to incorporate food groups and dietary ingredients—including whole grains—may help align quantitative nutrient-density metrics with the evolving food and nutrition policy.

## Notes

This study was funded by Cereal Partners Worldwide and General Mills, Inc.

Author disclosures: AD is the developer of the Nutrient Rich Food (NRF) index, a nutrient-profiling model, and has received grants, contracts, and honoraria from entities, both public and private, with an interest in nutrient density of foods, meals, and the total diet. NM has received funding for investigator-initiated grants from General Mills Bell Institute of Health and Nutrition and serves as an unpaid scientific advisor on the Oldways Whole Grains Council. EB has received funding for presentations on whole grain by Quaker Oats and General Mills, Inc., and research funding from Cereals Partners Worldwide. She is a member of the working group of the Whole Grain Initiative. FV is employed by MS-Nutrition. JS is employed by General Mills, Inc. GM is employed by Cereal Partners Worldwide. CJS is retired and has no conflicts of interest to report; previously, he has received funding for investigator-initiated grants from Cereal Partners Worldwide and Kellogg's, manufacturers of whole-grain breakfast cereals. He is a member of the European Healthgrain Forum and the Definitions Working Group of the Whole Grain Initiative. All the other authors report no conflicts of interest.

Perspective articles allow authors to take a position on a topic of current major importance or controversy in the field of nutrition. As such, these articles could include statements based on author opinions or point of view. Opinions expressed in Perspective articles are those of the author and are not attributable to the funder(s) or the sponsor(s) or the publisher, Editor, or Editorial Board of *Advances in Nutrition*. Individuals with different positions on the topic of a Perspective are invited to submit their comments in the form of a Perspectives article or in a Letter to the Editor.

Abbreviations used: DGA, Dietary Guidelines for Americans; FNDDS, Food and Nutrient Database for Dietary Studies; HEI, Healthy Eating Index; HSR, Health Star Rating; LIM, Limit; NP, nutrient-profiling; NRF, Nutrient Rich Food (index).
